# A Review of the Evolution of Residual Stresses in Additive Manufacturing During Selective Laser Melting Technology

**DOI:** 10.3390/ma18081707

**Published:** 2025-04-09

**Authors:** Peiying Bian, Ali Jammal, Kewei Xu, Fangxia Ye, Nan Zhao, Yun Song

**Affiliations:** 1 Xi’an Key Laboratory on Intelligent Additive Manufacturing Technologies, Shaanxi Key Laboratory of Surface Engineering and Remanufacturing, Xi’an University, Xi’an 710065, China; 2 School of Materials Science and Engineering, Xi’an Jiaotong University, Xi’an 710018, China

**Keywords:** selective laser melting (SLM), residual stress (RS), formation mechanism, regularities of distribution

## Abstract

Residual stress (RS) is one of the main reasons for component failure during an additive manufacturing (AM) process, especially using selective laser melting (SLM) technology. This paper reviews RS’s investigation methods, formation mechanisms and regularities of distribution. When considering recent research progress, studies indicate that the dominant stress is primarily attributed to thermal stress induced by significant laser temperature gradients during the rapid melting and forming process, which subsequently transforms into RS upon cooling to room temperature, as verified by simulation and experiments. Then, the distribution regularities of RS are analyzed. SLM RS gradually increases when it is measured from the surface layer to the substrate. In the plane direction, at the center and edge of the part, tensile stresses are found; as for the middle area, which is the transition area of compressive stress, the whole plane stress remains in an equilibrium state. Based on the forementioned conclusions, the three-dimensional distribution diagram of RS on the sample was constructed. Finally, the strategic approaches for stress mitigation are briefly discussed. The excessive stress in forming can be reduced by process parameter matching, and the RS can be greatly remitted by pre-treatment/post-treatment, so as to improve the quality of formed parts. This review provides a valuable theoretical basis for practical applications of SLM.

## 1. Background

Additive manufacturing (AM) is an advanced technique for producing complex geometries and small production batches, including parts that are challenging to manufacture with conventional methods; the technique has been subjected to a tremendous amount of research and industrial interest in recent years [1]. Selective laser melting (SLM) is one of the most widely used metal AM techniques, utilizing a laser as an energy source to achieve high-quality and efficient fabrication [2]. However, the SLM process also results in large and anisotropic residual stresses (RS). RS can lead to deformation and failure in some cases, but under certain conditions, it may enhance mechanical properties, depending on grain orientation and size [3]. Some facts show that large residual stress can cause excessive deformation or production failure, which seriously affects the implementation of the process scheme in workpiece manufacturing [4]. Therefore, a thorough understanding of how RS forms and distributes is crucial for optimizing SLM process parameters and minimizing defects. Recently, many research institutes, including some teams, have analyzed and explored residual stress-related mechanisms from different perspectives and methods [5,6], yet a review encompassing this information is not yet available.

According to the current state-of-the-art research, there are mainly two approaches to study the residual stress for SLM: experimental and simulation methods. In one experiment, RS was quantified by different measuring instruments or methods, including multi-point measurement in the plane area and in-depth measurement after stripping. However, the measurement error and range of different methods are not enough to reach a solid conclusion [7,8]. The simulation method can analyze the thermal evolution process of its stress, from the previous static small-scale single-layer or single-channel simulation [9] to dynamic multi-layer and multi-channel simulation [10,11,12].These studies revealed that the thermal coupling response is more in line with the actual process characteristics [6,13].

Furthermore, for additive components, RS may be alleviated by post-process heat treatment; the development of large residual stresses in-process can lead to excessive distortion, which can cause production failure or errors [1]. In this review, the knowledge available in the literature regarding RS was assembled and analyzed through the combination of simulation and experimentation. From thermal stress generation, residual stress formation, regularities of distribution and the effect of RS in the SLM process are described in a clear and concise manner. Finally, the mitigation and control of RS are summarized and prospected; the magnitude and distribution of RS may be alleviated by post-process heat treatment or by adjusting the combination of process parameters. The aim of this review is to serve as a concise yet thorough reference for researchers, as well as to provide a technical reference for the industrial application of SLM technology.

## 2. RS Investigation Methods

As mentioned before, residual stress in SLM is typically analyzed using two approaches: experimental methods and simulations. The experimental method is measured by different measuring methods, including mechanical contact and non-contact sensing. Those methods can be used for multi-point measurement in the plane area and depth measurement after stripping. However, the accuracy and range limitations of these methods pose challenges for advancing research in this area. Simulation methods, which can analyze the thermal evolution process of stress, include the finite difference method (FDM), finite element method (FEM) and finite volume method (FVM) et al. The methods are developing from the previous static small-scale, single-layer and single-channel simulation to dynamic multi-layer and multi-channel simulations. Such studies have revealed that the thermal coupling response is more in line with the actual process characteristics.

### 2.1. Typical Experimental Method

1. Hole-drilling method

**Figure 1:**
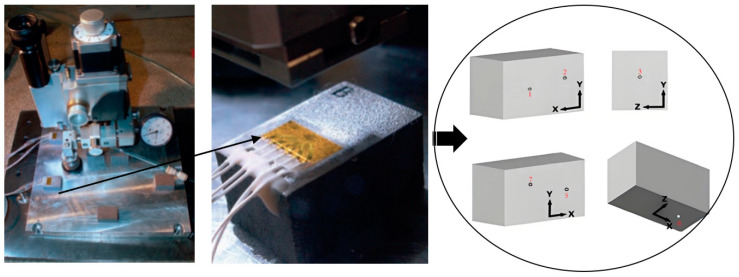
Measuring position and principle of hole-drilling method [14].

Despite being an invasive technique, hole-drilling (HD) measurements are widely considered accurate and reliable in industrial applications. The principle of the HD method is to drill a small hole in a stress field with a special drill bit, as shown in Figure 1. Usually, the diameter of the borehole is 2 mm and the depth is 1–1.5 mm [14,15]. If the balance of the stress is damaged, the stress around the hole will be readjusted. By measuring the elastic strain increment near the hole, the residual stress in the hole can be calculated using the principle of elastic mechanics according to the ASTM E837-13/20 standard [16]. The accuracy of the results of the small-hole method depends on the accuracy of the sticking position of the strain gauge. The smaller the aperture, the higher the accuracy of the relative position. In particular, the HD method has its advantages in measuring the distribution of stress along the depth [15]. Its measurement principle, using the stress release method, has been studied for several years, and the calculation accuracy is considered to be high. According to many references, residual stress data showed that specimens without support exhibit lower residual stresses in comparison to those with support [15,17]. Results also revealed that RS on the top surface was reduced when a substrate with no overhang was used [18,19,20].

2. X-ray diffraction measured

**Figure 2:**
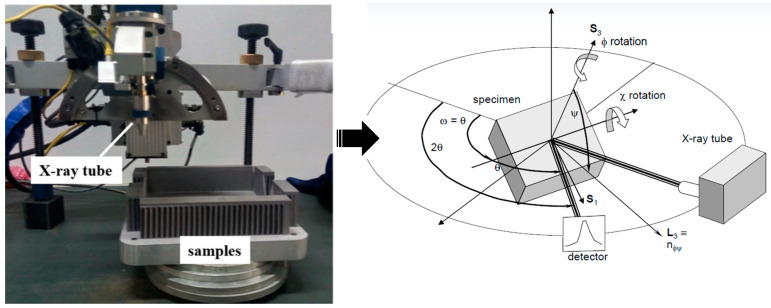
Measuring position and principle of X-ray diffraction method [28].

X-ray diffraction (XRD) measurement is a simple and efficient test method, as shown in Figure 2, but its measurement mechanism is complex; it is the most widely used method for RS [21,22,23]. In fact, the basic principle of X-ray diffraction measurement of residual stress is to measure the displacement of the diffraction line as the original data, and the measured result is actually the residual strain. Therefore, the residual stress is calculated from the residual strain using Hooke’s Law. When there is residual stress in the sample, the distance between crystal faces will vary. When Bragg diffraction occurs, the diffraction peak will change, and the moving distance is directly related to the stress [24,25]. The residual stress σ can be calculated by irradiating the X-ray at different incidence angles on the sample for several times; the calculation is carried out by measuring the corresponding diffraction angle 2θ and finding the slope M of 2θ to sin2ψ [26]. The XRD measurement method is mainly used to characterize the surface stress [27]. If the measurement is carried out along the depth, the corrosion method, electropolishing is required to strip the part for layer-by-layer testing [28].

3. Neutron diffraction measurement

**Figure 3:**
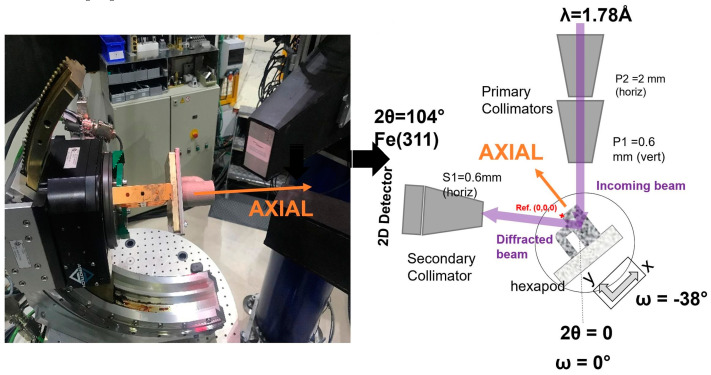
Measuring position and principle of neutron diffraction method [34].

Neutron diffraction (ND), as illustrated in Figure 3, is another widely used technique, particularly effective for measuring stress in thick and dense materials. The principle of the ND method is to employ the wave property of neutrons in order to obtain the RS information in the material by analyzing the scattering of neutrons. The neutron diffraction method is a very effective method to measure RS [29,30], it can measure the residual stress in some dense and thick material. It has also good adaptability to different material with different properties. In particular, it focuses on three-dimensional stress testing, which has good application prospects in engineering. Some valuable conclusions are obtained where, by increasing the layer thickness, the stress magnitudes and gradients are significantly reduced [31]. Non-destructive neutron diffraction was utilized to measure residual stresses and their distributions across wall thickness [32] and over cross-sections [33].

Furthermore, some other measurement means, such as crack compliance, incremental slitting, the ultrasonic method, and other complex methods to investigate residual stress, have also been reported. Such methods as the hole-drilling method combined with the X-ray diffraction method [19,35], and the hole-drilling method combined with simulation [20,36], and even X-ray-diffraction, hole-drilling, and the contour-method approaches [37] have been employed for RS measurement. The error of the hole-drilling measurements was found to be less than the XRD measurements (always less than 5% of the measured value) [37].

The residual stress testing literature for SLM forming is shown in Table 1. It shows that the data trends are similar but the data values of measurements widely vary. This also indicates that XRD, as a simple and efficient measurement method, has been adopted by many scholars and institutions, and some subsequent practical measurements in this paper mainly adopt this method.

### 2.2. Typical Simulation Methods

1. Plane stress calculation

**Figure 4:**
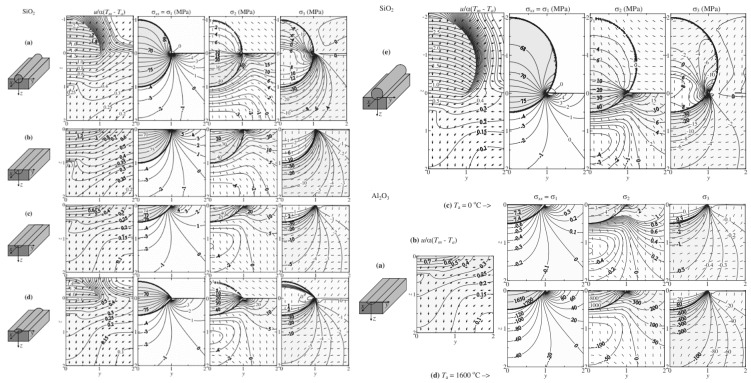
Plane stress distribution of simulation diagram [46].

Residual stress prediction has gained increasing attention from researchers, particularly through the use of the finite element method (FEM) [45,46]. Based on the discrete method of finite elements, the elastic mechanics of plane stress is solved, and the continuous regional stress distribution curve is obtained. The stress trend can be reflected by the density and the divergence direction of the curve in Figure 4 [46]. However, the current analysis of plane stress lacks sufficient clarity and intuitive representation, especially for three-dimensional (3D) distribution.

2. Three-dimensional stress simulation

**Figure 5:**
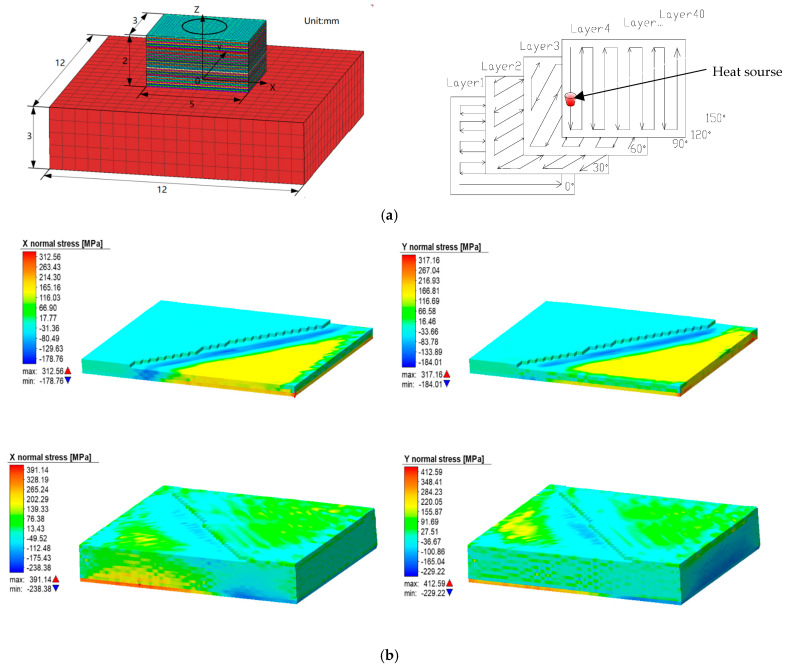
Three−dimensional stress distribution of simulation diagram. (**a**) Simulation modeling. (**b**) Results of residual stress [51].

With the development of finite element extension algorithms and the improvement of computing power, three-dimensional stress simulation with layer distribution has become a research hotspot [47,48]. The definition of the 3D simulation heat source, the design of the workpiece model, the setting of the process parameters and the setting of the calculation conditions have become more user friendly and efficient. The scope of simulations has expanded from single-layer and single-channel models [49] to multi-layer and multi-channel analyses, allowing for more accurate cyclic stress predictions [50], and the statistical change of stress over time has become more accurate, as shown in Figure 5 [51,52]. Hence, the stress variation with respect to layer thickness can now be well analyzed by simulation.

3. Comparison of simulation and experiment

In fact, the simulation method inevitably simplifies the environmental conditions for reflecting a complex multi-scale physical field of laser melting [53,54]. There are also many supporting studies for real verification of the simulation, as shown in Table 2. By comparing the simulation and experiment, a literature review shows that there may be some errors between simulation and actual measured values, but the general trend of stress evolution is consistent, which has good practical significance for correcting the finite element simulation model. Therefore, the simulation results trend is developing in the direction of high precision and high efficiency.

## 3. Main Research Results

### 3.1. The Formation of RS [58]

**Figure 6:**
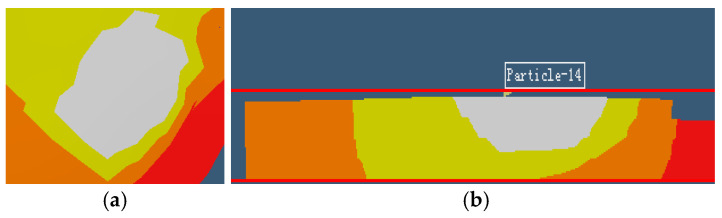
Topography of melting pool due to laser heating in SLM simulation. (**a**) Top view. (**b**) Section view [58].

**Figure 7:**
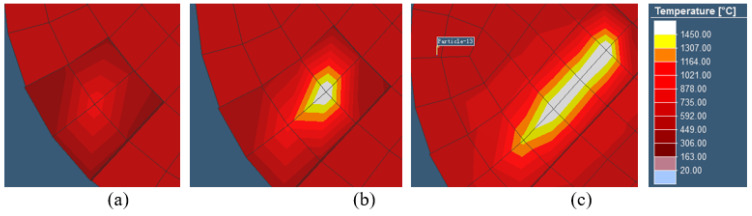
Development of melting pool of single track heating in SLM as the laser beam travels (**a**) 0.2 mm, (**b**) 0.3 mm, and (**c**) 0.6 mm [58].

According to the pyrogen model established by experimental analysis, the heat source is in accordance with the Gauss pyrogen model; the laser beam radiates heat from the center to the surrounding area. Due to the high intensity and concentrated energy of the laser, the temperature of the laser irradiation zone rapidly exceeds 2000 K, leading to the immediate formation of a molten pool with distinct length, width, and depth. The simulation results reveal that the molten pool exhibits an inverted conical shape, as shown in Figure 6a,b. As the laser beam travels, the molten pool evolves into a continuous molten pool strip during the forming process, as shown in Figure 7.

**Figure 8:**
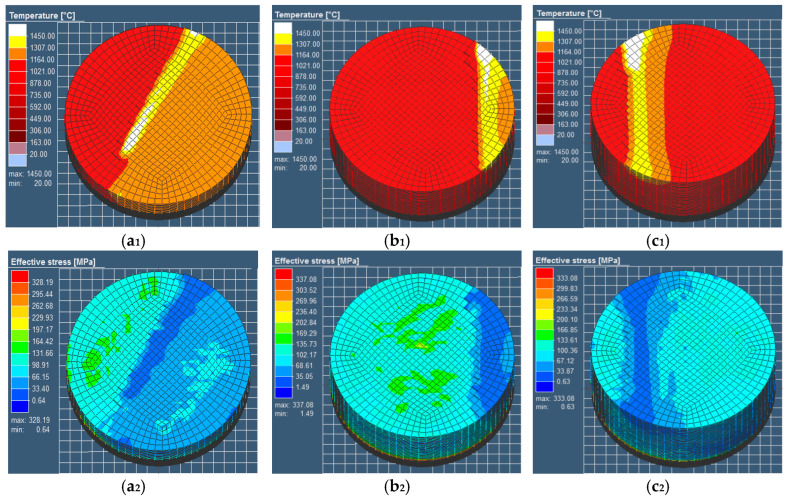
Temperature fields and equivalent stress distributions at simulation moments. (**a_1_**,**a_2_**) Tenth layer (t = 10 s). (**b_1_**,**b_2_**) Twenty-eighth layer (t = 33 s). (**c_1_**,**c_2_**) Thirty-ninth layer (t = 48 s) [58].

Figure 8 presents the temperature and stress history of representative points across different layers in the component simulation model, corresponding to the laser scanning path. As evident from Figure 6, the first layer initially exhibits ambient temperature conditions. Upon irradiation by the laser heat source, the temperature experiences a rapid increase, peaking at 2100 K—significantly above the material’s melting point. This instantaneous heating induces complete melting of the metal powder particles. Following laser source removal, the temperature precipitously drops below 1000 K, indicating an extreme thermal gradient. Subsequently, when a fresh powder layer is deposited and subjected to laser scanning, a molten pool forms and propagates into the underlying solidified layer. This thermal interaction between layers induces reheating of the previously deposited material through conductive heat transfer from the newly melted zone. As shown in Figure 8(a_1_–c_1_), the temperature of the laser source is continuously heated at different deposition heights. The pool size is very close to the geometrical size of the melting pool obtained in the experiment under similar conditions in the literature [61,62]. However, the magnitude of temperature fluctuations diminishes with successive layer deposition due to thermal attenuation effects. Note that the subsequent heating may produce a “micro annealing” effect of the underlying materials [58,63]. The oscillatory behavior dissipates as the process transitions to the pure cooling stage at 50 s, ultimately allowing the temperature to decay to ambient levels. Correspondingly, it can be observed from Figure 8(a_2_–c_2_) that the stress is very small when the sample is heated at the same moment as Figure 8(a_1_–c_1_), because the metal is in a molten state. After cooling, the residual stress of the bottom part is larger. The thermal attenuation cycles propagate uniformly through the material, with their characteristic number and penetration depth being governed by two key factors: the total number of overlying layers and the specific laser energy parameters employed.

**Figure 9:**
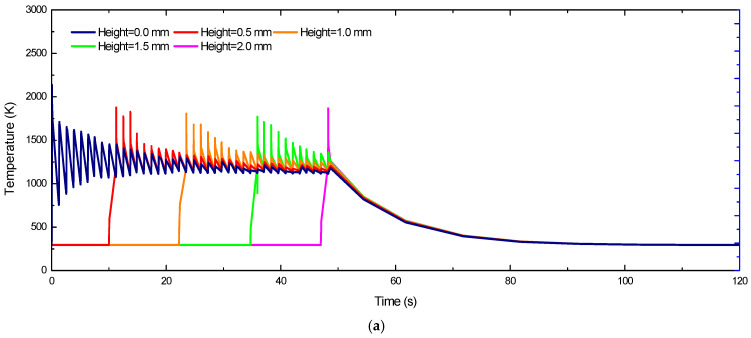

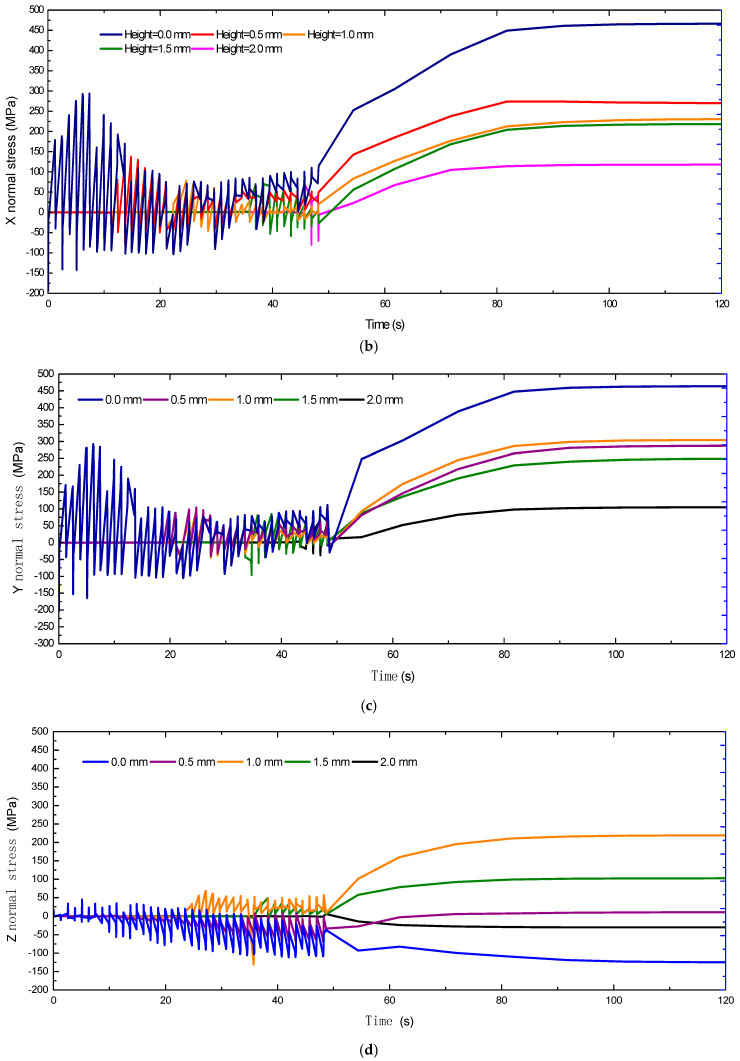
Temperature evolution curve and stress evolution curve in simulation. (**a**) Temperature. (**b**) X−stress component. (**c**) Y−stress component. (**d**) Z−stress component [58].

By referring to the coordinate system shown in the reference [58], the same position points near the center of samples of different layers in the same direction were selected. The thermal stress evolution curves in different directions are plotted, as shown in Figure 9.

According to the stress curves of the five layers tracked by the simulation, the X/Y stress has a similar evolution trend. In the process of laser layer-by-layer scanning, it is in an alternating state of tension and compression stress. After cooling to the final room temperature, the state of tensile stress changes into residual stress. Finally, with the change of layer thickness, the residual stress will also vary. The Z-direction stress is negligible in the laser scanning, and after stabilizing to room temperature, the RS will be in an alternating state between tension and compression, showing a relatively low and stable stress distribution.

### 3.2. The Evolution Mechanism of RS

**Figure 10:**
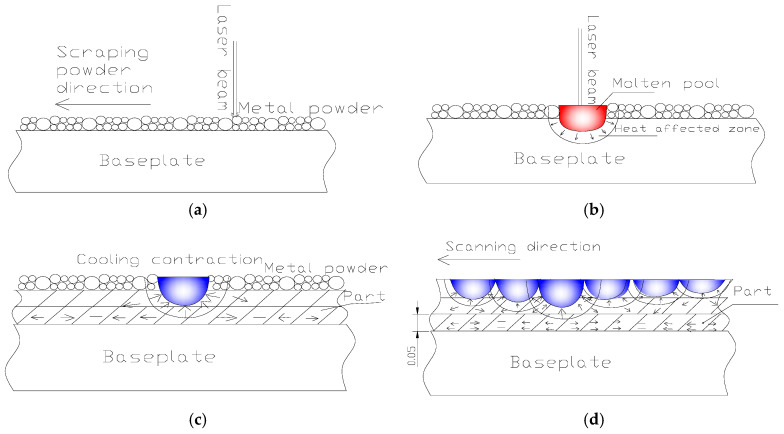
The evolution process of cyclic thermal stress in SLM. (**a**) Powder spreading. (**b**) Melting pool formation. (**c**) Solidification contraction. (**d**) Cyclic cooling (renewed from [58]).

Based on the simulation results, the process of stripe scanning a path in the same direction is analyzed as an example; the evolution of cyclic thermal stress throughout the deposition process is clearly demonstrated in Figure 10. The process comprises four distinct stages, as illustrated in Figure 10a–d. (a) The initial stage involves powder deposition, where the re-coater spreads fresh powder across the substrate while the laser system prepares to scan the new layer. (b) During the second stage, laser irradiation heats and melts the powder, forming a characteristic conical molten pool. (c) In the third stage, as the laser beam moves away, the molten material undergoes solidification. This process creates a thermal gradient where the periphery cools faster than the core, generating tensile stresses that radiate outward from the center. (d) The final stage occurs during subsequent layer deposition, where the newly formed molten pool thermally interacts with the underlying layer. The resulting heat transfer causes reheating of the previous layer, followed by directional solidification (edge-to-center) that further amplifies the tensile stress field.

Based on internal stress diffraction analysis, this study investigates thermally induced residual stresses generated during the SLM process. The inherent temperature gradient between heated and cooled regions results in non-uniform thermal expansion/contraction, thereby inducing significant thermal stresses. During rapid thermal cycling, atomic displacement caused by differential expansion may generate three distinct types of intrinsic stresses [64]: the first type is the macroscopic stress caused by different orientations and different spacing of grains; the second type is the pseudo-macroscopic stress produced by the inter-planar crystal spacing at similar grains; and the third type is microscopic stress by atomic motion, caused by crystal defects, vacancy, interstitial atoms and dislocation, etc. All three stress types (Type I macroscopic, Type II intergranular, and Type III microstresses) are inherently present in the SLM process. However, thermal–mechanical coupling analysis confirms that Type I macroscopic stresses dominate due to their system-scale influence on part distortion and residual stress accumulation. Additionally, phase-separated precipitates can induce transformation stresses during cooling and solidification, leading to non-uniform volumetric changes. These stresses, combined with thermal cycling effects, ultimately result in residual stress formation. The evolution of cyclic thermal stresses in selective laser melting (SLM) can be characterized by the following stages. First: The molten pool formation under laser irradiation increases atomic kinetic energy. Second: As the laser moves away, constrained atomic rearrangement during solidification creates tensile stresses due to crystallographic anisotropy and varying interplanar distances. Third: Subsequent heating–cooling cycles during multilayer deposition cause progressive stress attenuation [65]. Fourth: Temperature gradient-dependent solidification generates additional tensile stresses when cooling rates exceed heating rates [66]. Finally: The accumulated cyclic stresses converge to form the final residual stress state in the fabricated component.

### 3.3. The Regularities Distribution of RS

1. Residual stress along depth

**Figure 11:**
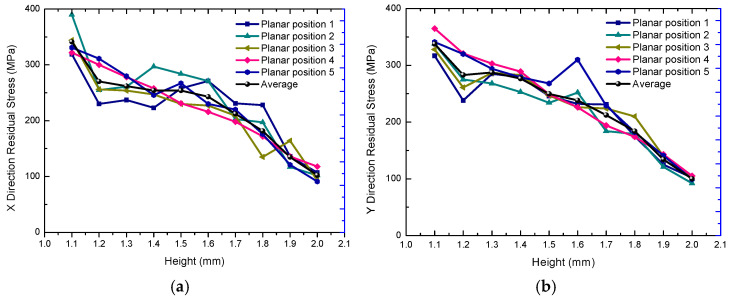
The simulated residual stress distribution along the build orientation shows (**a**) X−axis stress component and (**b**) Y−axis stress component.

**Figure 12:**
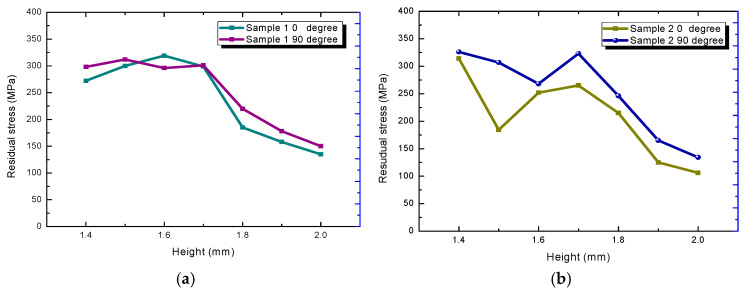
The residual stress distribution of two SLM specimens from XRD measurement, (**a**) sample 1, and (**b**) sample 2.

For the measurement of residual stress along the height direction, the simulation model and the experimental sample results were quantified simultaneously (Φ5 × 2 cylinder). In the simulation results, for the purpose of brevity, only the final RS distributions along the build direction from 1.1 mm to 2.0 mm height are shown, and can be seen in Figure 11a,b. For the experimental analysis, XRD tests were conducted on a total of seven layers of the deposition sample from the upper surface to the lower layer, as shown in Figure 12, from 2.0 mm to 1.4 mm. The results demonstrate a consistent decrease in tensile residual stresses along the build direction (Z-axis) for both X- and Y-oriented stress components, with the lowest stresses observed on the top surface [61,67]. Furthermore, residual stress measurements reveal strong correlations among multiple material points within the same layer.

2. Residual stress along plane direction

**Figure 13:**
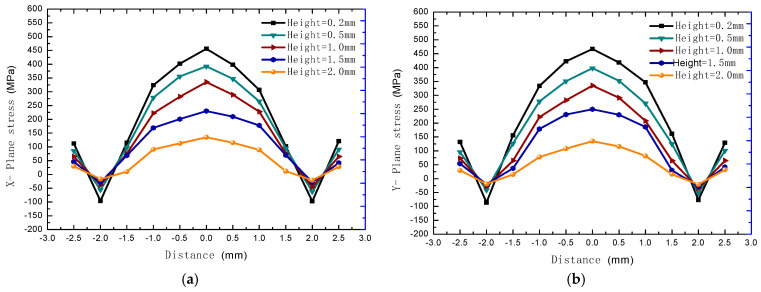
The plane residual stress distribution trend with forming height in simulation. (**a**) X−axis stress component and (**b**) Y−axis stress component.

**Figure 14:**
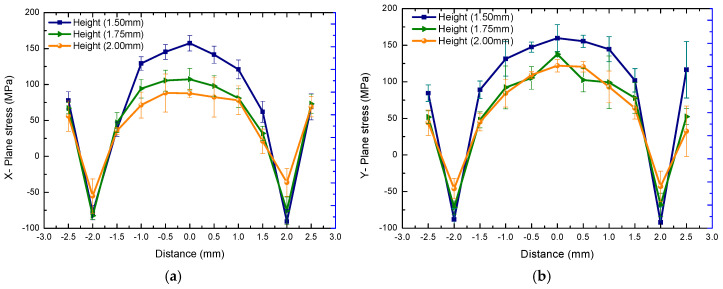
The plane residual stress distribution trend with forming height in experiment. (**a**) X−axis stress component. (**b**) Y−axis stress component.

Because the speed of laser motion is much higher than the cooling rate, a whole layer is cooled after melting close to the initial layer. Therefore, the cooling rate is higher in the edge than the middle position, and the molten material is cooled from the edge; the edge tension is formed by compressive stress, and the pressure is applied inwardly. Finally, the cooled center is subjected to tensile stress surrounding the resistance to contraction [68]. The stress variation trend diagram of the X-plane at the center section to the edge along the x-coordinate and the Y-plane stress at the middle section to the edge along the y-coordinate is formed as shown in Figure 13. Further, after the specimen is fully formed, it finally transitions from slow diffusion, and the outward tensile stress from the center changes to the compressive stress, then changes to the tensile stress at the edge. Finally, the whole plane stress reaches an equilibrium state. The same results were obtained when the test locations of the experimental samples were similar, as shown in Figure 14. In addition, due to the cyclic thermal effect of laser layer-by-layer scanning on the bottom layer, the plane distribution of residual stress in this layer is accumulated, and a trend of higher stress is also obtained as the measurement gets closer to the bottom layer. It can be seen that there are similar result trends at different layer depths.

## 4. Discussion

Residual stress is the internal stress that remains inside the material in the equilibrium state when no external force is applied. It is caused by non-uniform temperature change, non-uniform phase transformation and non-uniform plastic deformation in a machining process [69]. Laser forming has the characteristics of an extremely fast cooling rate. In a laser additive manufacturing heating process, as a result of different temperature distributions, melting and solidification are not synchronized during the cooling process; this will cause different parts of the expansion and contraction trend to be inconsistent, resulting in thermal stress [70,71]. At the same time, due to the inconsistency of the temperature distribution, the phase changes in different parts of the deposition-formed parts are not synchronized, the specific volumes between different phases will vary, and the phase transition and stress have a close correlation with each other during expansion or contraction [72,73,74].

### 4.1. Three-Dimensional Residual Stress Distribution

**Figure 15:**
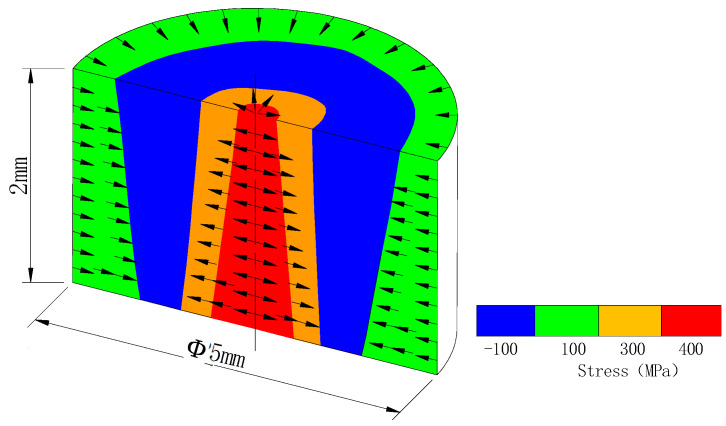
Three−dimensional residual stress distribution trend diagram of SLM-formed parts (i.e., material: 316LSS).

According to the above analysis of thermal field effects and surface stress depths of a layer-by-layer cycle, the distribution trend of thermal stress accumulated along the height and the evolution trend of plane stress from the center to the edge are obtained, as in the earlier cylinder experiment. The three-dimensional stress distribution diagram after SLM forming can be comprehensively analyzed from a three-dimensional model, as shown in Figure 15. In the plane layers, the cross-section diagram from the central part is used as a representation, and the plane stress at the position of each plane layer conforms to the equilibrium stress distribution trend of alternating tension and compression at each layer, as shown in Figure 13 and Figure 14. In the vertical direction, the accumulation of thermal stress makes the bottom stress greater than the top stress, as shown in Figure 11 and Figure 12. Hence, the plane stress is combined with the in-depth stress distribution to form a three-dimensional stress distribution trend diagram, such as the three-dimensional cylindrical simulation model. Therefore, the regional distribution and evolution process of the overall stress are clearly presented in Figure 15. Tensile stress is dominant at the core and edge of the sample, while compressive stress occurs in other transition regions. With the increase in the forming height, the tensile stress range decreases, and the compressive stress range increases; in particular, the blue compressive stress area changes with the forming height. The diagram accurately and comprehensively expresses the stress distribution effect of the formed specimen in its original state.

It should be noted that these results were obtained while the laser was scanning straight along the stripe. When the process parameters of SLM, such as laser power [75,76,77], scanning speed [78,79], overlap rate [80], layer thickness [81] et al. change, such changes will have a direct impact on the stress distribution. These results are also valid when different laser scanning paths [82], such as a checkerboard pattern, are scanned in the micro island region. Differences in the scanning area can only cause changes in the tensile and compressive stress areas depending on the scanning path [75,83] This explains why some literature measure the surface area as tensile stress [42,84,85] while others measure the surface as compressive stress [86,87]. Even in the same material with the similar process parameters, the measured residual stress values differ greatly, as shown in Table 2. Therefore, RS values at each position of the specimen after cooling differ greatly with a different cooling rate, a different cooling sequence and different process parameters. However, the existence of a large residual stress is inevitable due to the characteristics of laser processing technology [88,89,90]. As a result, RS adjustment in the SLM process is required to ensure the quality of a fabricated part.

### 4.2. Residual Stress Adjustment Method

1. Process parameter regulation

Recently, numerous studies have examined residual stress distribution in SLM-manufactured parts, such as significant improvements in microstructure and residual stress after the process parameters are regulated in a single or collective way; i.e., as the microstructure of the specimen is regulated [91,92,93], the mechanical properties are improved [94,95] and the residual stresses are reduced to a certain extent [96,97] by optimizing the process parameters of SLM forming. For instance, higher laser power [98] combined with lower scanning speed [99] generally results in increased residual stress levels. The key to process parameter regulation lies in achieving an appropriate balance of laser energy flux density [100]. Through experimental comparison or simulation research, the literature has some typical data cases in the process parameter matching for practical reference [99,101]. However, in order to further improve the quality of the SLM-formed specimens, it is also very necessary to carry out substrate preheating before printing and heat treatment regulation after printing.

2. Pre-treatment regulation

The pre-treatment of SLM forming by substrate preheating can effectively regulate the structural properties of the specimen [102,103,104]. Substrate preheating can reduce the cooling rate of the melt pool and coarsen the grains, thus affecting the microstructure and mechanical properties of the specimen. Furthermore, it can reduce the thermal stress caused by the temperature gradient, thus reducing the residual stress of the specimen [105,106]. However, preheating also increases the evaporation rate of the powder and the steam recoil pressure, thereby increasing the probability of pore formation [107], so it is very important to set an appropriate preheating temperature [108].

3. Post-processing regulation

As mentioned before, it is necessary to perform subsequent heat treatment on SLM-formed parts [26,109] for homogenizing the microstructure of the specimens, reducing internal defects, and eliminating residual stresses, thus reducing the anisotropy of the mechanical properties [110,111]. In addition, it has been proved that heat treatment can greatly improve the comprehensive mechanical properties [112]. Near the upper surface of the formed part, equiaxed grains are formed due to the rapid cooling. After heat treatment, the grains change to equiaxed grains. Heat treatment can gradually reduce and eliminate the anisotropy caused by the microstructure. With the increase in heat treatment temperature, the grain diameter at the side and top of the sample tends to be equal [113], the boundary of the molten pool gradually disappears, and the columnar crystal recrystallizes into equiaxed crystal [114,115].

4. Other adjustment methods

The hot isostatic pressing process (HIP) can lead the material to creep and plastically deform by applying high pressure in all directions to the specimen in a high-temperature environment, thus eliminating defects such as tiny pores and cracks that cannot be eliminated by ordinary heat treatment [116]. In addition, after a high-temperature solution treatment, the phase in the alloy can be transformed, thus further changing the mechanical properties of the specimen. In the future, some intelligent algorithms will also be applied to process allocation [117,118,119]. At the same time, several papers also carry out some reliable design research on the topology optimization of component ontology [120,121]. These methods will significantly affect the thermal distribution and residual stress in SLM forming, so as to continuously improve the comprehensive mechanical properties of SLM-formed parts.

## 5. Conclusions

This paper reviews recent research on residual stress in SLM, analyzing its formation mechanisms, influencing factors, and both experimental and simulation approaches. By combining simulation and experiment, the residual stress evolution processes of the SLM process are deeply researched. The main conclusions include:

- Residual stress formation: Due to the high laser energy input, the temperature gradient is high, which makes the hot-melt metal molecules displace and forms the thermal stress. With the conduction and radiation of the temperature field to the surroundings, the metal expands the crystallization and forms the orientation angle of the grain, which is retained as internal forces, and the thermal stress evolves into the residual stress.
- Residual stress detection: SLM residual stress measurement uses the experimental testing method and finite element prediction method. The stress values exhibit variations depending on material properties, processing parameters, and even the structure type. However, a consistent overall stress distribution trend is observed across all conditions.
- Residual stress distribution: The residual stress increases gradually from the surface layer to the bottom layer near the substrate in the vertical direction of the forming part. Meanwhile, the residual stress is distributed in the plane of the forming parts; at the center and edge, tensile stress is found; the compression plane in the middle is in a balanced state; the X and Y directions show basically the same distribution trend, and the Z-direction stress is negligible.
- Residual stress adjustment: The magnitude and distribution characteristics of residual stress can be effectively optimized through either pre/post-process heat treatment or strategic adjustment of processing parameter combinations.

## Figures and Tables

**Table 1:** Statistical table of residual stress test results.

Material	Model Size (mm^3^)	Layer Thickness (μm)	Power (W)	Scan Speed (mm/s)	σ_xmax_ (MPa)	σ_ymax_ (MPa)	σ_zmax_ (MPa)	Test Method	Reference
AlSi10Mg	30 × 20 × 10	30	120	900	----	----	0~320	HD	[17]
Ti6Al4V	30 × 30 × 10	75	150, 120	----	----	----	60~100	HD	[38]
X3NiCoMoTi18-9-5	15 × 70 × 12	50	200	450	200–379	220–447	200~400	ND	[39]
In625	22.5 × 50 × 1.25	30	125	500	−150–270	----	200~400	ND	[40]
316L SS	1 × 30 × 20	35	200	400	−64–54	−68–114	30~140	XRD	[41]
AlSi10Mg	250 × 250 × 250	30	400	500	10–140	10–150	----	XRD	[42]
Ti-6Al4V	12.7 × 12.7 × 10.1	50	713	78.8 J/mm^3^	----	----	197~360	XRD	[43]
AlSi10Mg	194 × 170 × 45	30	200	100	73	80	----	XRD	[44]

**Table 2:** Residual stress statistical table of simulation calculation compared with experimental investigation.

Material	Method	Sample Size (mm)	Layers or Tracks	Measuring Method /Mesh Size (μm)	Power (W)	Scan Speed (mm/s)	Thermal Stress or Residual Stress (MPa)	Reference
1.2709 tool steel	Experiment	15 × 70 × 12	240 layers, 50 μm/layer	ND	200	450	H: 200~391 V: 220~263	[39]
	FEM	15 × 70 × 10	10 layers, 1 mm/layer	10^3^	200	500	−120~360	
Ti-Ni Alloy	Experiment	1.19 × 0.315 × 0.0375	----	----	200	100	X: 57.5~105.7 Y: 44.3~110.5	[55]
	FEM		3 tracks, 1 layer 37.5 μm/layer	25 × 25 × 12.5	200	100	X: 7.5~82.5 Y: 9.5~84.5	
In 625	Experiment	18.13 R × 22.5 × 50	30 μm/layer	ND	125	500	−160~240	[40]
	FEM		600 μm/layer	----	125	500	−60~260	
Ti6Al4V	Experiment	30 × 30 × 10	3 Tracks, 75 μm/layer	HD	150, 200	----	60~100	[38]
	FEM	0.32 × 1.04 × 0.5	1 Track, 50 μm/layer	32.5 × 32.5 × 50	150, 200	----	TG simulates RS	
Ti6Al4V	Experiment	1.92 × 1.92 × 0.12	----	XRD	275	1100	400, 54, 358	[56]
	FEM		4 layers, 30 μm/layer	6 × 6 × 6	275	1100	1^#^: 492, 380, 316 2^#^: 235, 104, 15	
AlSi10Mg	Experiment	5 × 5 × 5	200 layers, 25 μm/layer	XRD	400	290	−92~−62	[57]
	FEM	1 × 5 × 0.075	3 layers, 25 μm/layer	20 × 20 × 25	400	290	−85~−58	
316L SS	Experiment	Φ5 × 2	50 μm	XRD	160	500	100~350	[58]
	FEM	Φ5 × 2	50 μm	100 × 100 × 50	160	500	100~420	
316L SS	Experiment	----	50 μm	----	200	700	----	[59]
	FEM	0.4 × 0.8 × 0.2	5Tracks, 100 μm/layer	30 × 30 × 15	200	700	X: −100~250 Y: −300~100	
Ti55531	Experiment	----	30 μm	XRD	140	600	830~878	[60]
	FEM	4×1×0.5	Single Track, 30 μm/layer	10 × 10 × 7.5	140	600	825, 840	

## Data Availability

The datasets used and analyzed during this study are available from the corresponding author on reasonable request.
